# Effects of Root Zone Aeration on Soil Microbes Species in a Peach Tree Rhizosphere and Root Growth

**DOI:** 10.3390/microorganisms10101879

**Published:** 2022-09-20

**Authors:** Maoxiang Sun, Xiaolong Liu, Kaiwu Shi, Futian Peng, Yuansong Xiao

**Affiliations:** State Key Laboratory of Crop Biology, College of Horticulture Science and Engineering, Shandong Agricultural University, Tai’an 271018, China

**Keywords:** root-zone aeration, peach, soil microbial, soil nutrient content, plant potassium-to-nitrogen ratio

## Abstract

The oxygen content in the root zone considerably affects the growth and development of peach trees. However, few studies have been conducted on the effects of the oxygen content in the root zones of peach trees on soil microbes and root growth. Four-year-old Ruiguang 33/*Prunus persica* (L.) Batsch trees were used to study the effects of root-zone aeration on soil microbes in a peach orchard, as well as on the soil nutrient contents, peach tree root systems, and plant potassium-to-nitrogen ratios. The results showed that the root-zone aeration substantially increased the soil oxygen content in the root zone and changed the soil microbial community structure. Compared with the control, the relative abundances of soil nitrogen-fixing microorganisms (*Beta proteobacteria and Bradyrhizobium elkanii*) and potassium-solubilizing microorganisms (*Bacillus circulans*) under the root-zone aeration conditions were greatly enhanced. Root-zone aeration increased the soil’s alkaline nitrogen content, available potassium content, and organic matter content, as well as the number and thickness of new white roots of peach trees, and root activity was increased significantly. At the same time, root-zone aeration changed the relative contents of total potassium and total nitrogen in the plants and considerably increased the potassium–nitrogen ratio in the shoots. The results indicate that aeration in the root zone can change the soil microbial community structure, increase the abundances of nitrogen-fixing and potassium-solubilizing microorganisms, and increase the plant potassium-to-nitrogen ratio, which are conducive to peach fruit quality.

## 1. Introduction

The peach, belonging to the family Rosaceae and the genus *Amygdalus* L., is an important deciduous fruit tree in China and one of the fruit trees with the largest cultivation areas and the highest yields in the world [1]. Root system management is the core of the management of fruit tree cultivation, and is the key to achieving high quality and high yield through rational root system management. Root systems are fundamental to plant growth as important organs that absorb water, nutrients, and oxygen [2]. Oxygen is essential for all aerobic organisms, and taller plants need oxygen to sustain metabolism and growth [3]. Hypoxia is often encountered throughout plant life, such as during sudden rainfall, flooding, or microbial activity. Rhizosphere hypoxia reduces plant growth through its effects on root function [4]. Changes in the oxygen environment of the root zone affect the respiration, growth, and development of root systems. The rhizosphere environment and root respiration are important factors in the normal growth of root systems [5]. The injection of air into the soil reduces soil oxygen deficiency and improves crop yields [6,7]. Nakano [8] and Xu et al. [9] reported that rhizosphere ventilation improved the gas environment in the tomato rhizosphere, thereby improving root activity and nutrient uptake. Wen et al. [10] reported that rhizosphere aeration enhanced the root activity of maize, increased the ability of crops to absorb water and nutrients, and promoted plant growth. Other studies have shown that aeration can alleviate anoxic soil conditions, increase crop yield [6,11], and increase the sugar content of sugar beetroot [12].

Soil microorganisms are not only an important part of soil, but also the main promoters of soil nutrient cycling. They play an important role in biogeochemical cycles, including soil organic matter decomposition, nutrient release, energy transfer, etc. [13]. Soil microorganisms participate in nutrient cycling, affect fertilizer-use efficiency, and directly or indirectly affect the physical, chemical, and biological parameters of the soil as indicators of soil changes [14]. Rhizosphere microbial activity contributes to increases in soil fertility through effects such as the exudation of soluble compounds, storage and release of nutrients, mineralization of nutrients, decomposition of soil organic matter, and dissolution of potassium [15,16,17,18]. Soil microorganisms serve as an indicator of soil health quality, and soil’s physicochemical properties can affect the characteristics of the soil microbial community [19]. Soil microbial activities affect the availability of soil nutrients and the absorption and utilization of those nutrients by root systems, ultimately affecting the growth and development of plants [15].

The oxygen environment in the root zone affects the root system architecture and its physiological activities, which affect the absorption and utilization of nutrients by the root systems. Xiao et al. [20] showed that root-zone aeration promoted the occurrence and growth of fine root systems of young peach trees and promoted plant growth. Potassium is the most sensitive element in response to the oxygen environment in the root zone; it is an indispensable element in plant life activities, and plants must absorb an appropriate amount of K^+^ from the soil for normal growth and development [21]. Potassium is a very important element in the growth and development of fruit trees and has a positive effect in enhancing their photosynthetic properties and promoting the transport of photoassimilates [22]. It is also involved in the synthesis and transport of sugar and starch, promotes the conversion of starch to sugar in fruits, and favors the increase in sugar content in the fruit [23]. The application of potassium to orchard soil is closely related to the growth and development of fruit trees and the formation of fruit flavor quality and yield, and soil potassium deficiency can reduce fruit flavor quality and affect the yield [24]. Guo et al. [25] showed that the application of potassium fertilizer can effectively improve peach fruit yield and quality. Nitrogen is an important element in the growth and development of plants and the formation of fruit quality. The application of nitrogen fertilizer can improve peach growth and increase yield, but excessive nitrogen supply can affect the absorption of other elements and decrease the quality of fruit. Li et al. [26] found that the quality of peach fruit decreases with an increasing nitrogen application rate. Therefore, proper nitrogen application is critical to fruit quality. Aerobic and anaerobic microbes are broadly distributed in soil [27], and changes in the oxygen environment will inevitably affect the diversity, composition, and structure of soil bacterial communities [28]. Previous studies have shown that soil aeration can effectively improve the numbers of soil microorganisms and soil enzyme activity [29,30]. Qian et al. found that aeration increases soil bacterial diversity and nutrient transformation under mulching-induced hypoxic conditions [31]. Li et al. found that artificial soil aeration increases soil bacterial diversity and tomato root performance under greenhouse conditions [32].

The root systems of peach trees have high respiratory intensity and large oxygen demand, and the oxygen content in the root zone considerably affects the growth and development of the peach trees [20]. However, the effects of aeration on the soil microorganisms in peach orchards, the relative contents of potassium and nitrogen (potassium–nitrogen ratio) in peach trees, and the soil nutrient content are not clear. For this reason, this study used 4-year-old Ruiguang 33/*Prunus persica* (L.) Batsch trees as the test materials, and the aim was to elucidate whether root-zone aeration could regulate soil nutrient content and plant potassium–nitrogen ratios by affecting soil microorganisms.

## 2. Materials and Methods

### 2.1. Experimental Sites, Plant Materials, and Sampling Time

**Figure 1:**
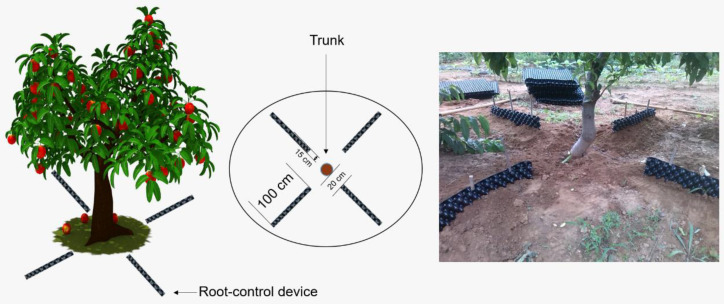
Root-zone aeration device and treatment schematic.

The experiment was carried out at the horticultural experiment station of Shandong Agricultural University with 4-year-old Ruiguang 33/*Prunus persica* (L.) Batsch trees as the test materials in March 2018 (117°16′ E and 36°17′ N). The growth conditions of the test materials were the same, and the basic physical and chemical properties of the tested soil were as follows: pH value of 6.74, alkaline hydrolyzable nitrogen content of 45.02 mg∙kg^−1^, organic matter content of 12.63 g∙kg^−1^, available phosphorus content of 35.24 mg∙kg^−1^, and available potassium content of 84.51 mg∙kg^−1^. The growing conditions for the peach trees were day and night temperatures of 31 and 23 °C, respectively, a natural photoperiod of around 12.5 h, and constant relative humidity of 30%. There were two treatments in the experiment: RV (root-zone aeration treatment), and CK (non-aeration as a control). A total of 20 replicates were set for each treatment and were randomly arranged. The experimental procedure was as follows: For aeration, radial furrows were excavated 20 cm from the trunk below the canopy, with an excavation depth of 40 cm, a width of 15 cm, and a length of 100 cm, and a folded root-control device with a height of 50 cm and a length of 200 cm was placed in the radial furrow. After the root-control device was placed in the radial furrow, three bamboo poles with a diameter of 1.5 cm and a length of 60 cm were inserted into the middle of the folded root-control device to ensure that a certain gap was retained in the middle of the root-control device after being folded, so that air could enter. Control radial furrows were excavated 20 cm from the trunk below the canopy, with an excavation depth of 40 cm, a width of 15 cm, and a length of 100 cm, and a folded root-control device with a width of 50 cm and a length of 200 cm was placed in the radial furrow. After the root-control device was placed in the radial furrow, the soil was directly backfilled into the gap to prevent air from entering (Figure 1).

The soil oxygen contents at horizontal distances of 20 cm from the aerator and vertical depths of 30 cm from the ground were determined on 18 March, 3 May, 18 June, 3 August, and 18 September 2018. On 19 September, the soil oxygen contents at horizontal distances of 10, 20, and 30 cm from the aerator and vertical depths of 10, 30, and 50 cm from the ground were measured. Rhizosphere soil samples were collected. On 20 September, five sites were randomly selected for each treatment to collect fresh soil samples at a horizontal distance of 20 cm from the aerator and a vertical depth of 30 cm from the ground (10 g per point, three biological replicates per treatment), which were then mixed; impurities—such as root systems, weeds, soil animals, and stones—were removed, and then the samples were passed through a mesh and mixed evenly for soil microbial analysis. On 18 March, 18 June, and 18 September 2018, soil samples were collected to determine the contents of soil alkali-hydrolyzable nitrogen, available potassium, and organic matter, and shoots and root systems were collected to determine the total nitrogen and potassium contents and potassium–nitrogen ratio of each organ. Three sites were randomly selected per treatment at each time point. On 4 October, the length (cm) and stem diameter (mm) of shoots of different treatments were determined, and the shoot stem diameter × 100/shoot length was calculated. On 15 October, the number and the diameter of new root systems were investigated; 3 sites were randomly selected for each treatment. The root systems were taken back to the laboratory, and the root system architecture parameters were determined using the professional WinRHIZO root–measuring system (Pro STD4800, Shanghai Zequan, Shanghai, China), including the total surface area (cm^2^), number of root tips (number), total volume (cm^3^), number of branches (number), number of intersections (number), mean root diameter (cm), and root system composition. The root systems were graded according to diameters of <2 mm, 2–5 mm, and >5 mm, and the total length (cm), total volume (cm^3^), and total surface area (cm^2^) of the different grades of root systems were determined. Every indicator was measured 3 or 5 times, and the average value was used.

### 2.2. Determination of the Soil Oxygen Content

The oxygen content in the soil was determined using a 13.05.04 Pro portable gas analyzer (AgriEco Apptec Shanghai LLC, Shanghai, China).

### 2.3. Determination of the Soil Nutrient Content

The soil organic matter content was determined by the potassium dichromate volumetric method (thermal dilution method) [33]. The soil’s available potassium content was determined by the NH_4_OAC extraction flame photometric method [33]. The soil alkali-hydrolyzable nitrogen was determined by the alkaline diffusion method [33].

### 2.4. High-Throughput Sequencing Method for the Microbial Community

Soil microbial total DNA was extracted from 0.25 g soil samples with an E.Z.N.A.^®^ Soil DNA Kit (Omega Bio-tek Inc., Norcross, GA, USA) according to the manufacturer’s instructions. The DNA concentrations were measured using a NanoDrop™2000 spectrophotometer (Thermo Scientific, Waltham, MA, USA). Polymerase chain reactions (PCRs) for N-transforming functional genes were performed as follows: 95 °C for 5 min, followed by 40 cycles of 94 °C for 45 s and 55 °C for 45 s, 72 °C for 1 min and, finally, 72 °C for 10 min. The analysis of the bacterial 16S rRNA gene and the fungal ITS region was performed on the Illumina MiSeq platform (www.i-sanger.com, accessed on 25 November 2021). The sequences of the 16S rRNA primers were V4 region 515F and 806R (515F:5′-GTGCCAGCMGCCGCGGTAA-3′, 806R:5′-GGACTACHVGGGTWTCTAAT-3′) [34]; the sequences of the ITS primers were ITS5-1737-F (5′-GGA AGT AAA AGT CGT AAC AAG G-3′) and ITS2-2043-R (5′-GCT GCG TTC TTC ATC GAT GC-3′) [35]. The PCR products were verified on 1% agarose gel and purified. The purified PCR products were subcloned into a PMD18-T simple vector (TaKaRa, Dalian, China), and the positive clones from each sample were randomly selected for sequencing (BGI, Beijing, China). The recovered sequences were subjected to a BLAST search in the NCBI database. All of the verified sequences were aligned using the DNAMAN program (Lynnon Biosoft, Pointe-Claire, QC, Canada). Subsequently, standard plasmid DNA was extracted, and thermal cycling and data analysis were conducted with a real-time PCR detection system using the LightCycler^®^ 480 Cycler (Roche, Mannheim, Germany) to estimate the abundance of anammox, AOA, and AOB, *amo A*, and the *nirS* and *nirK* genes. The sequencing of 16S rRNA and ITS was completed by Beijing Novogene Bioinformatics Technology Co., Ltd. (Beijing, China).

### 2.5. Determination of Indicators Related to Root System Architecture

Root system architecture parameters were determined using the professional WinRHIZO root-measuring system (image resolution of 4800 dpi), including total root surface area (cm^2^), total root volume (cm^3^), total root length (cm), and average root diameter (cm).

### 2.6. Determination of the Contents of Total Nitrogen and Total Potassium in Plants

The total nitrogen content of the plant samples was determined by the Kjeldahl method, and the total potassium content was determined by the flame photometric method [33].

### 2.7. Determination of Plant Growth Indicators

The length of the shoots (cm) was measured using a meter ruler, the shoot diameter (cm) was measured using a Vernier caliper, and the stem diameter × 100/shoot length was calculated.

### 2.8. Statistical Analysis

Two-way analysis of variance (ANOVA) followed by Duncan’s test was used to examine the differences between experimental treatments. Differences were considered significant at *p* < 0.05. The significance of the differences between samples was assessed by Duncan’s multiple range tests using SPSS version 20.0, with three technical replicates for each sample. In the beta diversity study, the weighted UniFrac distance and the unweighted UniFrac distance were used to measure the dissimilarity coefficient between two samples. The numbers on the upper side of the squares in the figure are the dissimilarity coefficients between the two samples. According to the species annotation and abundance information of all samples at the genus level, we selected the top 35 genera in terms of abundance and, according to the abundance information in each sample, clustered them at the species and sample levels, and drew a heatmap to find out which species were clustered more or less abundantly in which samples.

## 3. Results

### 3.1. Effects of Root-Zone Aeration on the Soil Oxygen and Nutrient Contents

**Figure 2:**
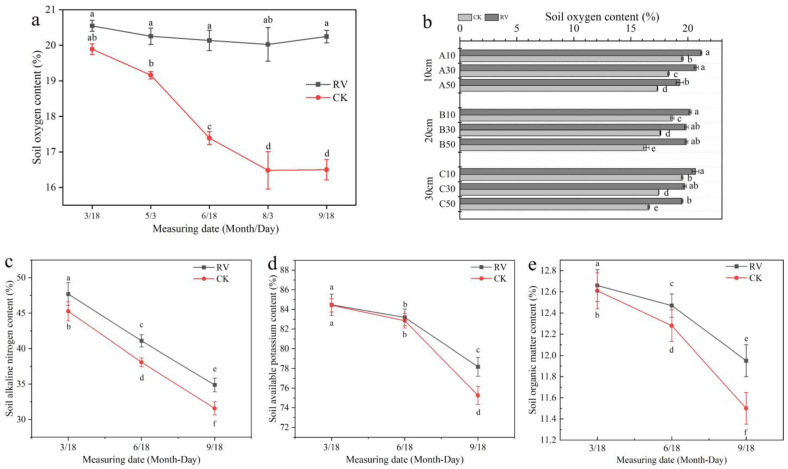
Effects of root-zone aeration on the soil oxygen content and soil nutrient content: (**a**) The soil oxygen contents at horizontal distances of 20 cm from the aerator and vertical depths of 30 cm from the ground at different times. (**b**) The soil oxygen contents at horizontal distances of 10, 20, and 30 cm from the aerator and vertical depths of 10, 30, and 50 cm from the ground; A, B, and C represent the horizontal distances of 10, 20, and 30 cm from the aerator, respectively. (**c**) Soil alkali-hydrolyzable nitrogen content. (**d**) Soil available potassium content. (**e**) Soil organic matter content. RV: root-zone aeration treatment; CK: non-aeration as a control. Each point represents the mean ± standard error (*n* = 3). Different lowercase letters indicate significant differences between treatments (*p* < 5%, two-way ANOVA).

It can be seen from Figure 2a that the oxygen supply in the soil treated with root-zone aeration was maintained at a high level, which was substantially higher than that of the control treatment. Figure 2b shows the effects of the root-zone aeration treatment on the soil oxygen content in the root zones (at different depths) with horizontal distances of 10 cm, 20 cm, and 30 cm from the aerator. The results indicated that the oxygen supply in the root zone under the root-zone aeration treatment was always maintained at a high level and was significantly higher than that of the control treatment. The farther away from the aerator in the horizontal direction, the lower the oxygen content of the soil; the deeper the soil layer in the vertical direction, the lower the oxygen content of the soil.

Root-zone aeration changed the soil nutrient status of the root zones of young peach trees. It can be seen from Figure 2c–e that the soil nutrient content in the peach tree root zones decreased throughout the whole growing season, and the difference between the two treatments gradually increased. The aeration in the root zone increased the soil alkali-hydrolyzable nitrogen content, the soil available potassium content, and the soil organic matter content, and the differences were significant.

### 3.2. Effects of Root-Zone Aeration on the Alpha Diversity Index, PCA Plot, and Species Abundance Clustering Heatmap

**Figure 3:**
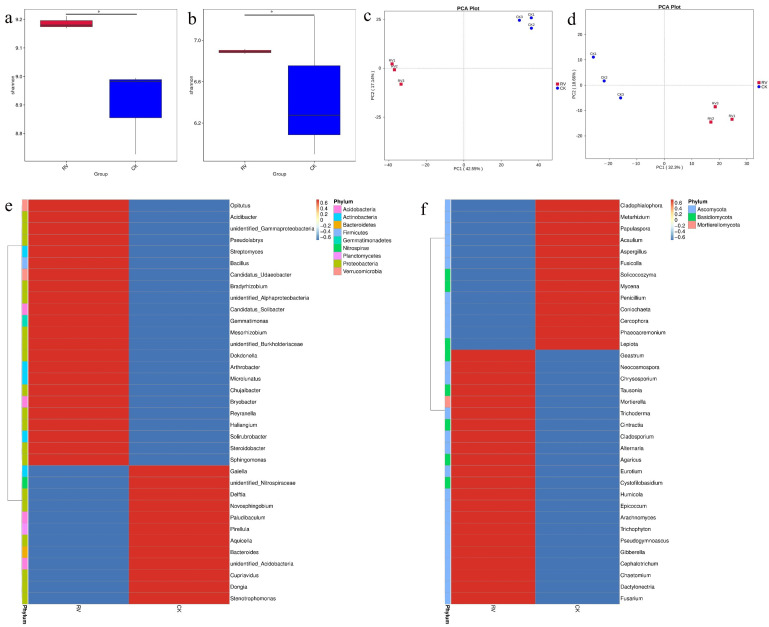
Effects of root-zone aeration on the alpha diversity index, PCA plot, and species abundance clustering heatmap: (**a**) Bacterial Shannon index. (**b**) Fungal Shannon index. (**c**) PCA at the genus level for bacteria. (**d**) PCA at the genus level for fungi. (**e**) Genus-level bacterial species abundance clustering heatmap. (**f**) Genus-level fungal species abundance clustering heatmap (* *p* ≤ 0.05).

In the analysis of differences between groups in the alpha diversity index, the boxplot can intuitively reflect whether the differences in species diversity between groups are significant. The Shannon index is one of the indices for estimating the diversity of microorganisms in a sample; the higher the value of the Shannon species index, the greater the species richness of the sample. As can be seen in Figure 3a,b, the species richness of the RV treatment was higher than that of the CK treatment in terms of bacterial communities and the fungal communities. The smaller the dissimilarity coefficient between the two samples, the smaller the difference in species diversity. From Supplementary Figure S1a,b, it can be seen that the RV treatment was significantly different from the control treatment, indicating that the root-zone oxygenation changed the soil microbial community structure. Principal component analysis (PCA) is an applied variance decomposition based on Euclidean distances. The more similar the community composition of the samples, the closer they are in the PCA plot. As shown in Figure 3c,d, there were differences between the RV and CK treatments in terms of bacterial and fungal communities, and the same treatment had better repeatability. The abundance of soil bacteria and fungi was significantly different after RV treatment; the fungal species were mainly *Ascomycota* and *Basidiomycota*, and the bacterial species were mainly *Proteobacteria*, *Acidobacteria*, and *Actinobacteria*. (Figure 3e,f). At the genus level, after RV treatment (and after removal of unidentified strains), *Chujaibacter, Bacillus*, *Bradyrhizobium*, *Pseudolabrys*, *Bryobacter*, *Microlunatus*, and *Reyranella* were the dominant bacterial genera (Figure 3e), while *Mortierella* and *Trichoderma* were the dominant fungal genera (Figure 3f).

### 3.3. Effects of Root-Zone Aeration on LDA Effect Size and Genus-Level t-Test

**Figure 4:**
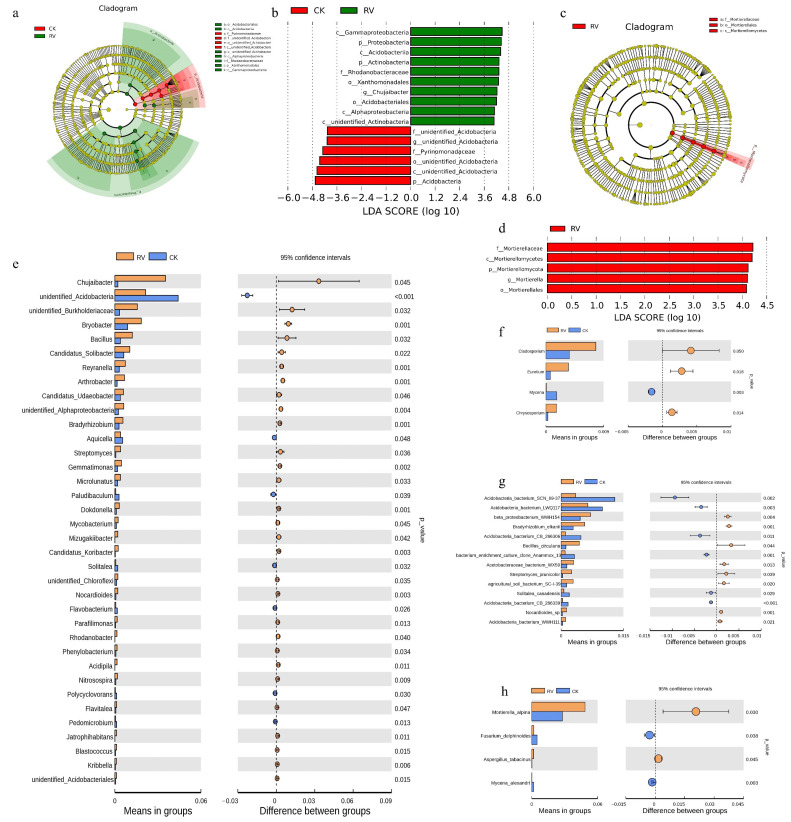
Effects of root-zone aeration on LDA Effect Size and genus-level *t*-tests: (**a**) Bacterial LEfSe clade map. (**b**) Bacterial LDA value distribution histogram. (**c**) Fungal LEfSe clade map. (**d**) Fungal LDA value distribution histogram. (**e**) Bacterial differential species abundance. (**f**) Fungal differential species abundance. (**g**,**h**) Significant differences in soil microbial counts at the species level.

LEfSe (LDA Effect Size) is an analytical tool for the discovery and interpretation of high-dimensional biomarkers that can be used to compare two or more subgroups. It emphasizes statistical significance and biological correlation, and is able to find biomarkers with statistical differences between groups. The statistical results of LEfSe include LDA value distribution histograms and evolutionary clade diagrams. In a clade diagram, the circles radiating from the inside to the outside represent the taxonomic level from phylum to genus (or species). As shown in Figure 4a,b, *c_Acidobacteriales*, *c_Acidobacteriia*, *c_Alphaproteobacteria*, *f_Rhodanobacteraceae*, *o_Xanthomonadales*, *c_Gammaprpteobacteria*, and *p_Proteobacteria* were the dominant bacterial species under RV treatment. As shown in Figure 4c,d, under RV treatment, *f_Mortierellaceae*, *o_Mortierellales*, and *c_Mortierellomycetes* were the dominant fungal species. In order to find the differences in species between groups at the genus level, a *t*-test was performed between groups to determine the species with significant differences (*p* value < 0.05). From Figure 2e, it can be seen that the species abundance of *Chujaibacter*, *Bryobacter*, *Bacillus*, and *Bradyrhizobium* in the RV treatment was significantly higher than in the CK treatment at the bacterial genus level, while the species abundance of *unidentified_Acidobacteria* was significantly lower than that in the CK treatment. From Figure 2f, it can be seen that the species abundance of *Cladosporium*, *Eurotium*, and *Chrysosporium* in the RV treatment at the fungal genus level was significantly higher than that in the CK treatment, while the species abundance of *Mycena* was significantly lower than that in the CK treatment. Figure 4g,h show significant differences in soil microbial counts at the species level: *Beta proteobacteria*, *Bradyrhizobium elkanii*, *Bacillus circulans*, *Acetobacteraceae_bacterium_WX59*, *Agricultural_soil_bacterium* and *Mortierella alpina* were significantly more abundant than in the control group, while the anammox bacteria *Bacterium_enrichment* was lower than in the control group.

### 3.4. Effects of Root-Zone Aeration on Genus-Level Evolutionary Trees and Functional Annotation of Relative Abundance

**Figure 5:**
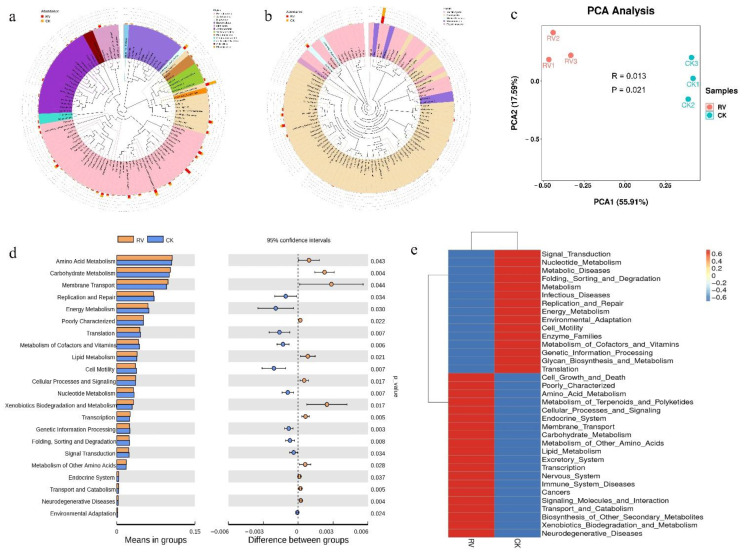
Effects of root-zone aeration on genus-level evolutionary trees and functional annotation of relative abundance: (**a**) Bacterial genus horizontal evolutionary tree. (**b**) Fungal genus horizontal evolutionary tree. (**c**) Functional prediction of PCA. (**d**) Bacterial functional annotation of relative abundance. (**e**) Bacterial functional annotation clustering heatmap (color scale: −0.6 to 0.6 abundance increases).

In order to further study the phylogenetic relationships of species at the genus level, the representative sequences of the top 100 genera were obtained by multiple sequence alignment. As can be seen from Figure 5a,b, compared with the control, the bacterial abundances of *Proteobacteria*, *Acidobacteria*, *Bacteroidetes*, *Actinobacteria*, and *Ascomycota* after root-zone aeration showed significant differences at the genus level. After aeration treatment, the oxygen environment in the root zone changed, and aeration increased the abundance of aerobic bacteria (*Nocardioides*), *Bacillus*, *Reyranella*, *Mortierella*, and *Fusarium*, and reduced the relative abundance of *Acidobacteria* and *Solicoccozyma*. From the functional prediction of PCA in Figure 5c, it was found that there were significant differences in soil bacterial function between the RV and CK treatments. Based on the bacterial database annotation results and the functional information on level 2 samples, functional *t*-test plots and heatmaps were generated, respectively. As shown in Figure 5d,e, compared with CK, the RV treatment played a positive role in amino acid metabolism, cell growth and death, cellular processes and signaling, lipid metabolism, and other processes.

### 3.5. Effects of Root-Zone Aeration on the Root Growth and Root System Architecture of Peach Trees

**Figure 6:**
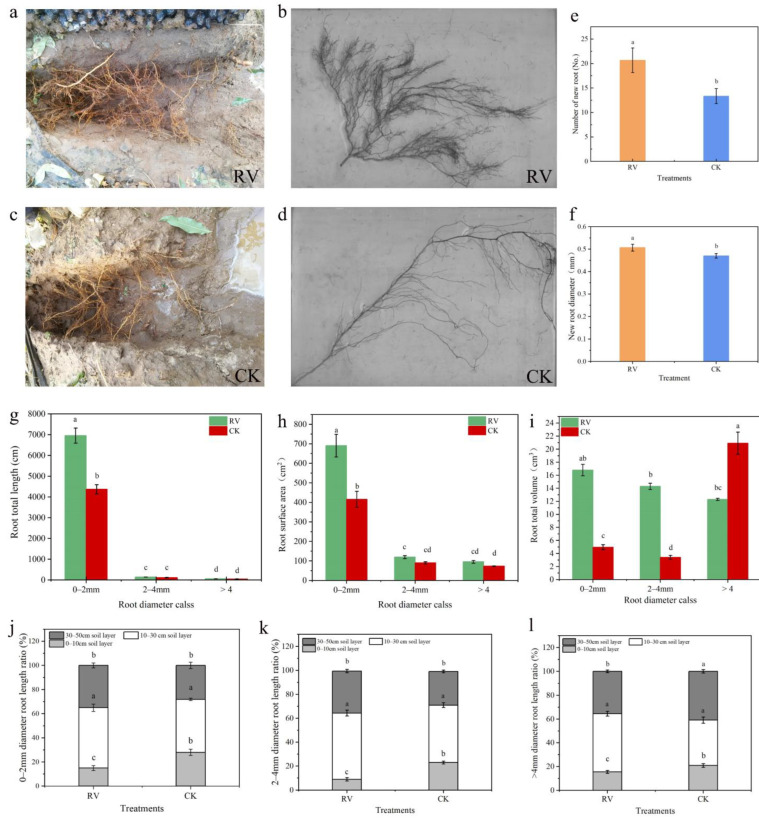
Effects of root-zone aeration on the root growth and root system architecture of peach trees: (**a**) Root system under root-zone aeration treatment. (**b**) Root system scan image under root-zone aeration treatment. (**c**) Root system under control conditions. (**d**) Root system scan image under control conditions. (**e**) Number of new roots. (**f**) Diameter of new roots. (**g**) Roots’ total length. (**h**) Roots’ total surface area. (**i**) Roots’ total volume. (**j**) Distribution characteristics of root systems with diameters of 0–2.0 mm in the vertical direction. (**k**) Distribution characteristics of root systems with diameters of 2.0–4.0 mm in the vertical direction. (**l**) Distribution characteristics of root systems with diameters of >4 mm in the vertical direction. Each point represents the mean ± standard error (*n* = 3). Different lowercase letters indicate significant differences between treatments (*p* < 5%, two-way ANOVA).

As can be seen in Figure 6a–d, root-zone aeration considerably promoted the occurrence and growth of peach roots, and large numbers of white new roots were observed. In the soil treated with root-zone aeration, there were more branches of the peach roots, the number of growing points was obviously increased, and a large number of network roots appeared. In addition, the new roots were thicker, and the lifespan of such a root system may be longer. In the control treatment, the number of peach root systems was low, the roots were fine and slender, and the life span of such roots may be shorter. As can be seen from Figure 6e,f, compared with the controls, the number and diameter of new roots of peach trees under the root-zone aeration conditions increased by 55% and 7.8%, respectively, and the differences were significant. As shown in Figure 6g–i, the root-zone aeration treatment substantially increased the total length, total surface area, and total volume of the root system with a diameter of 0–2 mm; compared with those of the control, the length, surface area, and volume of the roots in the soil treated with root-zone aeration increased by 59.03%, 66.26%, and 239.19%, respectively, and the differences were significant. It can be seen from Figure 6j–l that under the root-zone aeration treatment, the proportions of the root systems of peach trees with diameters of 0–2.0 mm and 2.0–4.0 mm in the 10–30 cm and 30–50 cm soil layers increased, and the proportion of the root system with a diameter > 4 mm in the 10–30 cm soil layer was greater than that of the control. This indicates that root-zone aeration promotes roots’ thickening and growth to deep soil.

### 3.6. Analysis of Root and Twig Growth’s Correlation with the Genus-Level Microbial Community

**Figure 7:**
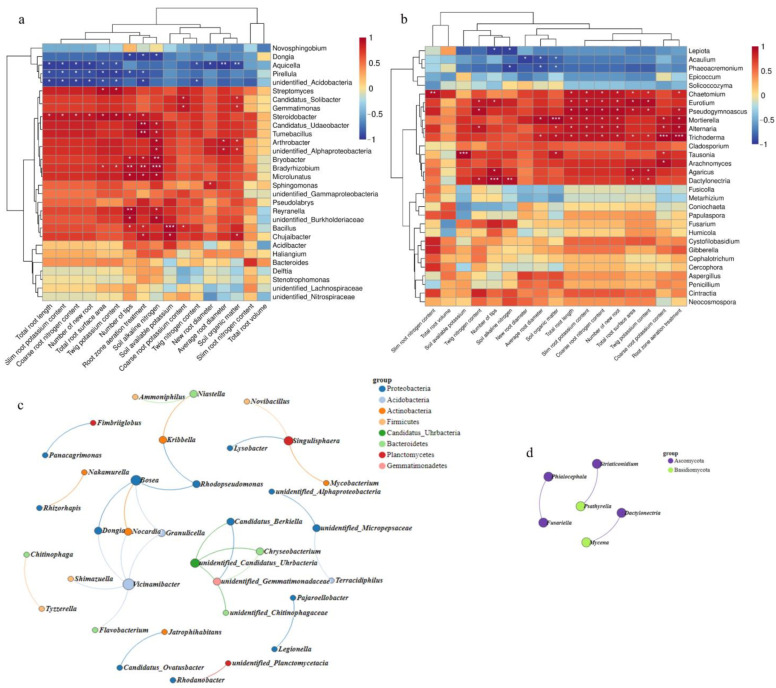
Analysis of root and twig growth’s correlation with the microbial community: (**a**) Analysis of root and twig growth’s correlation with bacteria. (**b**) Analysis of root and twig growth’s correlation with fungi. (**c**) Bacterial dynamic network diagram. (**d**) Fungal dynamic network diagram. * *p* ≤ 0.05, ** *p* ≤ 0.01, *** *p* ≤ 0.001; the scale represents the correlation, and the larger the |r| value, the stronger the correlation.

As can be seen from Figure 7a, the soil available potassium content was positively correlated with the abundance of *Bacillus* sp. Soil alkaline nitrogen content was positively correlated with the abundance of *Bradyrhizobium* sp. and *Bryobacter,* sp., and negatively correlated with *Dongia* sp. As can be seen from Figure 7b, soil available potassium content was positively correlated with the abundance of *Pseudogymnoascus* sp. and *Alternaria* sp. Soil organic matter content was positively correlated with the abundance of *Mortierella* sp. and negatively correlated with *Acaulium* sp. and *Phaeoacremonium* sp. Soil available potassium content was positively correlated with the abundance of *Tausonia* sp. Number of tips was positively correlated with the abundance of *Dactylonectria* sp. and negatively correlated with *Lepiota* sp. It can be seen from Supplementary Figure S2 that the abundance of *Mortierella* sp. was significantly positively correlated with the abundance of *Bacillus* sp., *Bradyrhizobium*, and *Microlunatus*, and significantly negatively correlated with the abundance of *Dongia* sp. and *Pirellula* sp. It can be seen from Figure 7c that *Vicinamibacter* sp. closely interacted with other genera after RV treatment. Figure 7d shows the correlation at the fungal genus level.

### 3.7. Effects of Root-Zone Aeration on Potassium and Nitrogen Uptake and the Potassium–nitrogen Ratio in Peach Trees

**Figure 8:**
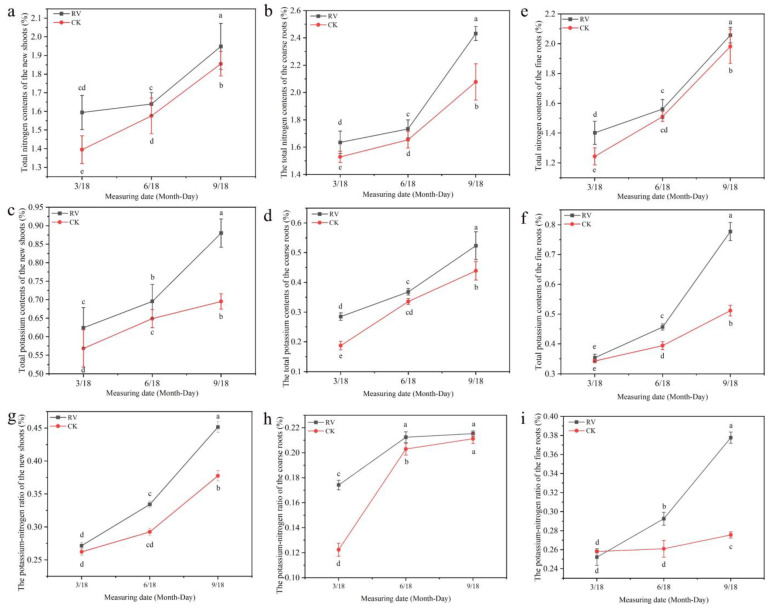
Effects of root-zone aeration on total potassium and total nitrogen contents in peach trees: (**a**) Total nitrogen contents of the new shoots. (**b**) Total nitrogen contents of the coarse roots. (**c**) Total nitrogen contents of the fine roots. (**d**) Total potassium contents of the new shoots. (**e**) The total potassium contents of the coarse roots. (**f**) Total potassium contents of the fine roots. (**g**) The potassium–nitrogen ratio of the new shoots. (**h**) The potassium–nitrogen ratio of the coarse roots. (**i**) The potassium–nitrogen ratio of the fine roots. Each point represents the mean ± standard error (*n* = 3). Different lowercase letters indicate significant differences between treatments (*p* < 5%, two-way ANOVA).

This study found that root-zone aeration treatment dramatically increased the nitrogen content and potassium content of peach trees. It can be seen from Figure 8a–f that compared with those of the control, the total nitrogen contents of the new shoots of the time points with root-zone aeration treatment were increased by 14.3%, 4.02%, and 5.05%, respectively; the total nitrogen contents of the coarse roots were increased by 6.93%, 4.75%, and 17.03%, respectively; the total nitrogen contents of fine roots were increased by 12.73%, 3.34%, and 3.79%, respectively, and the differences were significant; the total potassium contents of peach shoots were increased by 9.82%, 7.18%, and 26.59%, respectively; the total potassium contents of coarse roots were increased by 52.13%, 9.62%, and 19.30%, respectively; and the total potassium contents of fine roots were increased by 3.19%, 15.81%, and 51.82%, respectively, and the differences were significant. It can be seen from Figure 8g–i that the aeration in the root zone changed the relative contents of total potassium and total nitrogen in the peach trees, along with the potassium–nitrogen ratios of the new shoots, coarse roots, and fine roots. The potassium–nitrogen ratio of the shoots subjected to the root-zone aeration treatment was considerably increased.

## 4. Discussion

The root system is the link between the plant and the soil. A healthy soil environment can promote the development of the root system which, in turn, promotes the growth of plants [36]. The results of this study showed that root-zone aeration could maintain the soil oxygen content throughout the growing season at levels substantially higher than that of the control (Figure 2a,b). The root-zone aeration changed the soil nutrient contents in the root zone; the alkali-hydrolyzable nitrogen content, the soil organic matter content, and the available potassium content increased (Figure 2c–e); at the same time, the soil microbial species changed significantly (Figure 3). This may be because the soil nutrient contents are related to the soil microbial activity, soil enzyme activity, and nutrient uptake of plant roots. Previous research has found that under root-zone aeration treatment, changes in the soil gas environment take place, and the concentrations of O_2_ and CO_2_ in the soil change, altering the metabolic activity of microorganisms [37,38]. An increase in the soil oxygen content promotes the metabolic activities of aerobic microorganisms, accelerates the decomposition and conversion of soil organic matter, and increases the contents of soil’s available nutrients [7]; these observations are similar to the results of this study.

Microorganisms (e.g., bacteria and fungi) have a functional relationship with plants, interact with plants as a whole system, and play an important role in plants’ growth and development. Beneficial microorganisms promote plant growth and increase plant yield [39]. Biological nitrogen fixation, performed by prokaryotes—mostly bacteria—with the ability to convert atmospheric nitrogen into ammonia and, thereafter, other nitrogen compounds that can be assimilated by plants, stands out as a key process for agricultural production and environmental sustainability [40]. *Bradyrhizobium* [41] is an important genus of bacteria for biological nitrogen fixation and a member of the ‘rhizobium group’ of bacteria that establish mutualistic relationships with legumes (Fabaceae family), on which they induce nodules that are formed on roots [42]. However, some species and strains of *Bradyrhizobium* have been found to be capable of free-living nitrogen fixation [43,44,45]. Evidence has been found that *Bradyrhizobium* spp. may play a role in free-living N2 fixation (FLNF) associated with non-leguminous plants, without the formation of nodules—for example, in rice [46,47,48], maize [49], sweet potato [50], sorghum [44,51], and sugarcane [52,53]. *Bradyrhizobium elkanii* is of agricultural importance because it fixes atmospheric nitrogen [54]. Chen et al. [55] showed that β-proteobacteria can be specific symbionts of legumes, and also confirmed the role of β-proteobacteria in symbiotic nitrogen fixation in legumes. The measurement results showed that the relative abundances of *Bradyrhizobium elkanii* and *Beta proteobacteria* were significantly higher than those in the control treatment (Figure 4) and were significantly correlated with soil alkaline nitrogen (Figure 7a), indicating that soil aeration may enhance *Bradyrhizobium elkanii* and *Beta proteobacteria* activity, thereby increasing the soil content of alkali-hydrolyzed nitrogen.

The bacteria that are involved in the solubilization of potassium from K-bearing minerals are called potassium-solubilizing bacteria (KSB). These bacteria have the ability to convert insoluble/mineral K into available K in soil [17]. KSB in the soil and rhizosphere plays a central role in the cycling of K [56]. Shaaban et al. [57] found that feldspar inoculated with *Bacillus circulans* significantly improved leaf K content, fruit weight, yield, and fruit quality. Zeng et al. isolated a potassium-solubilizing strain *Bacillus Circulans* Z_1–3_ from soil. The potassium-solubilizing ability of the strain Z_1–3_ can increase solubilization of potassium by about 70% compared to the control experiments under the appropriate conditions. The strain Z_1–3_ was demonstrated to be an efficient potassium-solubilizing bacterium with broad potential application [17]. This study found that the contents of available potassium in soil increased after RV treatment, which was significantly correlated with the abundance of *Bacillus* (Figure 7a), which may because *Bacillus circulans* converts insoluble/mineral potassium in soil into available potassium that plants can absorb and utilize. Through the correlation heatmap (Figure 7b), we also found that the abundance of *Mortierella* was positively correlated with soil organic matter content, the abundance of *Trichoderma* was positively correlated with coarse root potassium content, and the abundance of *Dactylonectria* was positively correlated with the number of root tips. Root-zone aeration increased soil nutrient content by changing microbial species, which was beneficial to peach tree growth.

Studies have shown that high gas permeability is favorable for root growth [58]. Guo et al. [59] studied the effects of rhizosphere ventilation on the growth and root activity of potted maize and revealed that rhizosphere ventilation could increase root activity and improve plant growth. The study by Li et al. [60] showed that the root fresh weight, root dry weight, and root activity of cucumbers treated with root ventilation were substantially increased. Rhizosphere aeration can improve the root environment of peach trees, ensure aerobic respiration, increase nutrient uptake by roots, and promote plant growth [3]. In this experiment, the soil texture was clay loam, the gas permeability was poor, and the oxygen content in the soil became one of the factors limiting root growth. The results showed that an increase in the oxygen content of the soil promoted the occurrence of peach root systems, and the total root length, root area, root volume, and number of root tips were increased greatly (Figure 6), which increased the number of new roots and the root activity. The number of new roots under the root-zone aeration treatment was relatively high, which improved the lifespan of the root system and increased the root activity. The increase in the number of fine roots enhances the ability of plants to absorb nutrients, and the total surface area of the root system is closely related to the exchange and absorption of the root system and is a reflection of the passive absorption capacity of roots [61]. Moreover, aeration treatment in the root zone is favorable for the peach trees’ root systems to grow into the deeper soil layers, which is essential for improving the stability of the root systems and promoting their absorption and utilization of water and nutrients.

Bai et al. [62] showed that the application of potassium fertilizer could increase the intrinsic quality of peach fruit to different degrees and significantly increase the contents of soluble solids and soluble starch in the fruits. Potassium plays an important role in peach fruit quality and yield. This study indicated that rhizosphere aeration could promote the total nitrogen and total potassium contents in plant shoots, coarse roots, and fine roots compared to those under the control treatment (Figure 8). In combination with the plant potassium-to-nitrogen ratio (Figure 8), it can be seen that under aeration conditions the potassium content in the plants was relatively increased, promoting the transport of photoassimilates and the conversion of starch in the fruit to sugar, which is beneficial for increasing the sugar content in fruit. The above results show that root-zone aeration can regulate the nitrogen and potassium balance of plants and has a positive effect on improving fruit quality.

## 5. Conclusions

In summary, root-zone aeration considerably increased the soil oxygen content in the root zone and changed the soil microbial community structure, significantly increasing the relative abundances of soil nitrogen-fixing microorganisms (*Beta proteobacteria* and *Bradyrhizobium elkanii*) and the potassium-solubilizing microorganism *Bacillus circulans*. The root-zone aeration enabled the root system to grow into the deep soil, and the number, diameter, and activity of the new white roots of the peach trees treated with root-zone aeration were greatly increased. Root-zone aeration changed the relative contents of total potassium and total nitrogen in the plant and considerably increased the potassium–nitrogen ratio of the shoots, and the shoots were plump. These findings indicate that root-zone aeration can increase the soil oxygen content, change the soil microbial community structure, increase the relative abundances of nitrogen-fixing and potassium-solubilizing microorganisms in the soil, increase the plant potassium–nitrogen ratio, and improve peach fruit quality.

## Data Availability

Not applicable.

## Supplementary Materials

The following supporting information can be downloaded at: https://www.mdpi.com/article/10.3390/microorganisms10101879/s1, Figure S1: Heatmap of soil microbial beta diversity index under different treatments—(a) bacterial beta diversity index; (b) fungal beta diversity index. Figure S2: Fungal and bacterial correlation heatmap.
