# Supplementary figures and images for: Effects of Root Zone Aeration on Soil Microbes Species in a Peach Tree Rhizosphere and Root Growth
Source: Microorganisms. 2022 Sep 20;10(10):1879. doi: 10.3390/microorganisms10101879 (PMC9611397; doi:10.3390/microorganisms10101879)

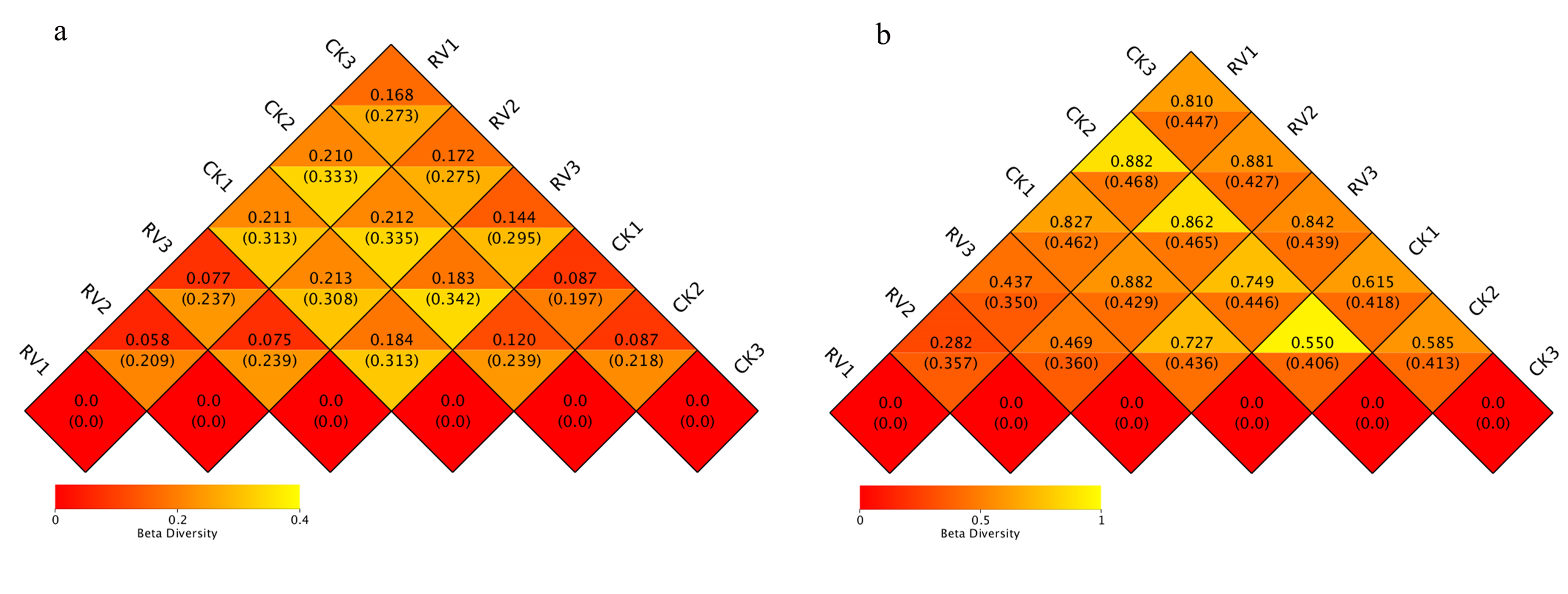

Supplement: Supplementary file 1 [file microorganisms-10-01879-s001.zip › Supplementary Figure/Supplementary Figure S1a and S1b.jpg]

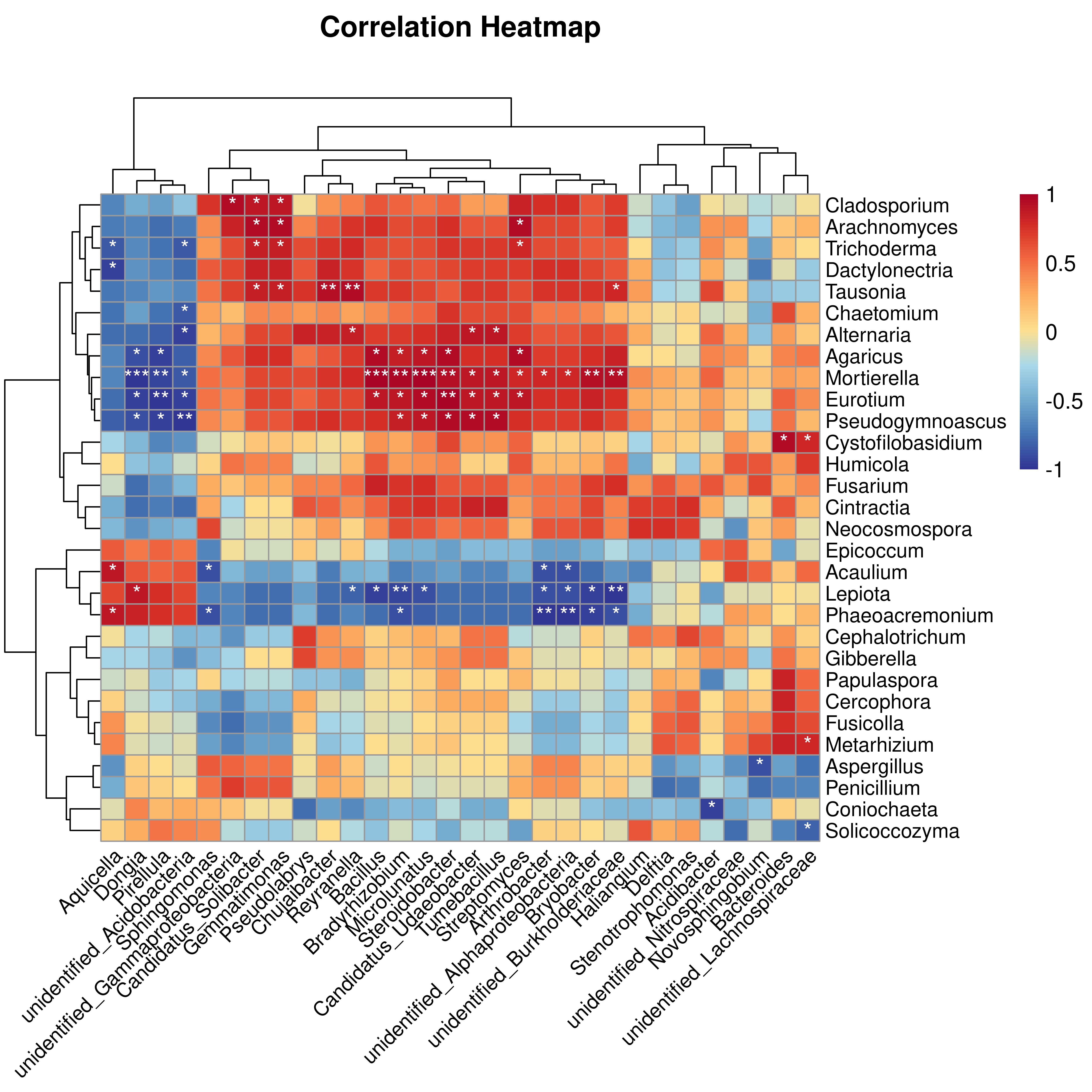

Supplement: Supplementary file 1 [file microorganisms-10-01879-s001.zip › Supplementary Figure/Supplementary Figure S2.jpg]
